# Critical appraisal of the european commission roadmap towards phasing out animal testing: navigating sociotechnical barriers to the regulatory acceptance of NAMs

**DOI:** 10.1016/j.namjnl.2026.100145

**Published:** 2026-10-10

**Authors:** Aleksandra Čavoški, Laura Holden, Robert G. Lee

**Affiliations:** Birmingham Law School, University of Birmingham, Edgbaston, Birmingham, B15 2TT, UK

**Keywords:** Chemical risk assessment, Animal testing, EU roadmap, New approach methodologies

## Abstract

The gradual rate of phasing out animal use in regulatory testing has been attracting greater attention from policy makers, the scientific community, and industry. In this paper we analyse the extent to which the strategic ‘Roadmap Towards Phasing Out Animal Testing for Chemical Safety Assessments’ of the European Commission is able to address sociotechnical barriers that have thus far hindered the greater regulatory acceptance of NAMs, including: the lack of consensus when applying scientific developments to value-laden risk assessments that have inherent uncertainties; the lack of stakeholder participation; regulatory culture; and routes to assurance. The roadmap is predominantly focused on the technical aspects of the transition, leaving the social nature of barriers to be addressed in greater detail in the implementation phase. A review of legal frameworks underpinning the roadmap identifies that more extensive statutory interpretation, particularly regarding the use of Weight-of-Evidence, would aid regulatory acceptance of NAMs. The paper critiques the short-, mid-, and long-term actions of the roadmap, and notes the absence of consideration of the EU judicial system, which plays an important role in providing interpretation to EU chemical law and its enforcement, in particular the obligation to use animal testing as a last resort. Although setting a clear final intention, this paper notes that with the longer-term goal to replace the use of non-protected life stages and invertebrates, and without hard milestone dates by which to indicate progress, the roadmap as a strategy is only able to provide limited clarity that NAM innovators and investors seek.

## Highlights

• The roadmap is predominantly focused on the technical aspects rather than social.
• Consensus and awareness raising, training, and an EU route to assurance will help.
• Legislative instruments do largely have the capacity to accept NAM data.
• The roadmap fails to stipulate hard phase out dates by which to indicate progress.
• The longer-term goal to phase out invertebrates may be problematic for innovators.

## Introduction

Despite an early legal commitment to deploy animal testing as ‘a last resort’, progress towards replacement and reduction of animal testing has been painfully slow.^1^ According to the latest data, ‘between 2015 and 2023, over 15 million animals have been used for testing for regulatory purposes in the EU, with almost 40% of them for chemical safety assessments.’^2^ Progress has been made with the development of non-animal methods, especially with the use of new approach methodologies (NAMs) such as organ-on-chip technology. Over time, the gradual phasing out of animal testing attracted greater attention of policy makers, the scientific community, and industry. No less important was the engagement of civil society, which was instrumental in encouraging the European Commission to produce a roadmap as a mark of commitment to the phasing out of animal testing for chemical safety assessments, in a noteworthy testament to the effectiveness of the ‘Citizens’ Initiative’ prescribed by Article 11 of Treaty on the European Union (TEU).^3^ The ‘Roadmap Towards Phasing Out Animal Testing for Chemical Safety Assessments’ (hereafter: ‘the roadmap’) was finally published in June 2026 and this article provides reflections on the main steps in the transition from traditional animal testing methods.

The main objective of the roadmap is to gradually ‘reduce all animal testing for chemical safety assessments across the EU’, provided that non-animal testing can provide the equivalent level of protection as the testing methods currently used.^4^ The plan of action is underpinned by a three-pillar structure, where each pillar focuses on one key theme that will assist in gradual transition. Pillar I predominantly focuses on legal and regulatory issues that will facilitate the transition. Pillar II echoes the EU commitment to stay at the forefront of research and innovation, as a key element for developing and refining non-animal testing methods. Finally, Pilar III recognises a need for concerted action by key stakeholders at national, EU, and international level.

This paper begins by analysing the extent to which the roadmap considers sociotechnical barriers that to date have hindered the greater regulatory acceptance of NAMs. We consider technical elements to be both science and law, but also the processes and procedures used to establish and deliver facets such as safe spaces. These elements, however, are not only technical but also have social aspects. Safe spaces, for example, must be developed such that they feel ‘safe’ for industry, meaning they must be made to feel assured and the forums perceived as neutral and open to enable meaningful discussion. Similarly, training may be a technical enterprise whereby scientific method is conveyed, but its social nature should not be overlooked - differently perceived trainer / trainee relationships should be considered; delivery preferences and needs should be understood; and expectations clear as to whether an event provides knowledge-deficit awareness raising or whether the output is to truly raise competence, which again can be fraught with social issues regarding participation and assessment. We consider the roadmap to have strongly considered the technical elements required of ‘what’ the path to transition may entail, but we consider that it remains relatively (albeit not completely) quiet on ‘how’ the implementation phase is to unfold.

We consider four main areas where barriers are identified: first, the need for consensus when applying scientific developments to value-laden risk assessments with inherent uncertainties; the need for stakeholder participation, in particular NGOs to garner societal acceptance; the current legislative interpretation and fora for engagement, as framed by regulatory culture; and routes to assurance. The paper presents a socio-legal analysis of how chemical legislation is currently implemented, followed by a consideration of the legal frameworks that underpin the roadmap on the possibility of a more extensive interpretation of current legislative instruments, to aid the regulatory acceptance of NAMs. This includes discussion of the significance of the ‘One Substance, One Assessment’ agenda; the short-, mid-, and long-term actions identified in the roadmap; the capacity of legislative instruments to accept a weight-of-evidence approach to data submissions; and the role of EU courts and the ECHA Board of Appeal. Finally, the paper considers the trajectory of transition, particularly in light of the longer-term goal of the roadmap to replace the use in testing of currently non-protected life stages and invertebrates, and the limited remaining term of this Commission, which therefore restricts its ability to set firm future dates.

## Sociotechnical barriers and the roadmap solutions

Our research to date has attempted to identify barriers to the full implementation of the 3Rs.^5^ The gradual phasing out of animal testing has been recognised as not being a linear process that follows the science but one that has been inhibited and affected by wider technical and social barriers to the transition.^6^ The roadmap does acknowledge the social feature of this transition, although the European Commission Staff Working Document (SWD), which reflects the preparatory work in building the roadmap, appears to focus primarily on the technical rather than the social barriers.^7^ Nonetheless, the roadmap addresses some of the most pertinent barriers discussed as part of Pillar I and Pillar III of the roadmap, in an attempt to allow better identification of steps for overcoming them.

### Scientific development

The roadmap recognises that NAMs have struggled to gain a regulatory foothold for ‘complex endpoints’, with ‘different information’ in comparison to animal testing being gleaned from NAMs, which can entail ‘molecular level’ measurements.^8^ We can similarly observe from case law that animal tests provide observational data, such as organ weight measurements, rather than information on the biological pathways (as provided by NAMs) to such harmful outcomes.^9^ The roadmap considers this a technical barrier, requiring a ‘new scientific framework’ that must be ‘developed and agreed’ to help deliver an equivalent level of protection.^10^ The roadmap is, however, light on the social challenge that securing this agreement will entail.

Currently, information about decision making of residual risk is difficult to attain, and, although the SWD enlarges on this process, it acknowledges that ‘the quantitative benchmarks for “acceptable” or “tolerable” need to be defined on the policy/risk management level’.^11^ Determining the severity of affect across a diverse population and the likelihood of occurrence across a potential range of uses and exposures is a huge task in terms of building consensus. The SWD does acknowledge, through reference to the work of Chiu and Paoli, that most chemical risk assessments do ‘lack any quantitative characterisation as to the likelihood, incidence, or severity of the risks involved’, but a step is potentially missing in addressing this self-proclaimed ‘mission impossible’ for a future NGRA framework.^12^ Such a quantitative assessment is not currently available from which to compare any human or environmentally-based NAM assessment. In comparison with analyses in other sectors and jurisdictions (such as pharmaceutical interventions or even safety of the built environment), considerations of the value of a statistical life and the societal value of risk reduction are rarely openly discussed in toxicology.^13^ Without providing quantitative dose-response data, these equations and comparisons become problematic, demonstrating the particular importance of developing quantitative *in vitro* to *in vivo* extrapolation (QIVIVE) and population exposure considerations. Discussions regarding levels of protection remain value-laden rather than objectively fixed. Designations of ‘high’ and ‘low’ risk and uncertainty may be sufficient at stages of screening and prioritisation, but not when regulators consider NAM data in the processes surrounding the more detailed evaluation of safe use and the authorisation or restriction of substances.

Considerations around levels of protection are likely to create a role for the ‘Roadmap Steering Team’ during and beyond the implementation period. However, an appreciation by the NAM development community of the forward use of their data in decision making would aid their understanding of user needs when developing models and help to ensure useability beyond prioritisation and into full risk assessment.

The approaches discussed in the SWD suggest a ‘relative’ or an ‘absolute’ strategy to demonstrate that protection is adequate.^14^ However, the ‘relative’ strategy suggests that comparisons are made with ‘traditional assessments’, which typically means animal studies. The roadmap contends that these have greater imperfections as a model for humans than NAMs, and so this ‘relative’ strategy would not achieve scientific betterment. Additionally, reference is made in the SWD to the use of the UN Globally Harmonised System of Classification and Labelling of Chemicals (GHS) for ‘conclusive’ hazard classification, however previous empirical research identified that EU regulators consider the Classification, Labelling and Packaging System (CLP, based on the UN GHS) to be restrictive and associated with recourse to animal studies.^15^ CLP, similarly to the REACH regulation,^16^ does allow a Weight-of-Evidence (WoE) approach that does not necessarily use animal studies; although reliance on NAMs will require an acknowledged reinterpretation of the text, as well as significant soft law guidance for support. Although the weaknesses of animal models are recognised in the SWD, the perceived limitation of CLP receives less attention, yet the legislative frameworks can form a (technical) barrier to the greater uptake of NAMs. That being said, the EU has shown to be willing to diverge from the UN GHS as it deems necessary: the EU has added unique hazard classes, such as for endocrine disruption, in divergence from the GHS. However, such deviations then sit in contrast to EU (and typically industry) support for international standardisation (such as the EU support for developing OECD TG, as is discussed further below in the 'Validation and Standardisation' section.). This juxtaposition reflects the difficult position of the European Commission between responding quickly to the European Citizens’ Initiative by developing a progressive roadmap, yet seeking to reduce trade friction for industry and maintain international consensus (and which, in turn, solidifies confidence).

In contrast, the ‘absolute’ strategy, based on probabilistic risk metrics, reflects approaches in other regulatory risk management settings, and from which precedents can be taken.^17^ Beyond the industrial chemical sector, different approaches have developed to manage risk; for example, international standards for phytosanitary measures have been developed to enable and manage global movements of food and crops by understanding parameters such as levels of detection, confidence, and efficacy. As noted in the SWD, ‘It may also be argued that probabilistic methods more adequately reflect the reality of potential chemical effects on populations’, which is a development on the existing tenfold assessment factors to deal with uncertainty. 18 Additionally, the SWD recognises that such an ‘absolute’ strategy is transparent and standardised and can be used where there is ‘sparse data’; a critical point where a risk assessment may otherwise resort to animal studies to create new data. As the SWD notes, such measuring of uncertainty would be a ‘superior’ quality of risk assessment, in contrast to the current paradigm.^19^ Regulatory approaches that can demonstrably minimise the over or under regulation of substances are optimal and therefore desirable in a risk-based system.

Whatever approach is taken by the Roadmap Steering Team, the European Commission ‘Catalogue of Transitional Initiatives’ will allow for a better oversight of existing initiatives and developments.^20^ Bearing in mind the vast and disparate research projects, a continuously updated repository to consolidate NAM information is necessary, not least to enhance transparency.^21^ However, there is a rich yet opaque landscape of initiatives across NAM development, training platforms, funding schemes, and collaborative networks. Obtaining information due to limited online visibility, coupled with under-representation of in-house industry initiatives, makes comprehension challenging and onerous. This has implications for the NAM development community to identify gaps in knowledge generation and to direct the provision of support, including the appropriate direction of future funding. The SWD notes that a task of the Roadmap Steering Team will be to promote the catalogue,^22^ and both the value and the difficulty of that task should not be underestimated. Encouraging stakeholders to engage thus far appears to have been troublesome, with the catalogue currently standing at around 35 initiatives; well short of the over 150 initiatives that our research identified. This difference raises the need for clarity as to the defined scope of the catalogue, and to consider what encouragement can be given for the catalogue to become and remain up to date, for example by linking the requirement for submission with bids to European Commission funding, or to organise submission at the point of publication or when seeking validation. Additionally, for clarity, we recommend a systematic approach that aligns initiatives with the short-, medium-, and long-term structure of actions within the roadmap.^23^

### Societal acceptance

The roadmap recognises the need to work together with an extensive group of stakeholders. This is predominantly the theme of Pillar III, which provides suggestions as to next steps. However, it is already clear from an analysis of Pillar I measures that this wider engagement results from several important factors. One is certainly the time that is needed to develop the scientific maturity of NAMs, followed by time required to achieve consensus of stakeholders. The roadmap also emphasises the requirement to ‘deliver a level of protection equivalent to that of currently established methods, as core principle of this roadmap.’^24^ Although the roadmap acknowledges that developing new risk assessment approaches will involve the ‘broad participation of regulators and stakeholders,’^25^ the journey to gaining this consensus remains complex and uncharted.

Also recognised in the roadmap is the need for understanding how change is to be managed across stakeholders, which requires ‘bespoke engagement.’^26^ There is a significant value to this approach. The first being that there is broad consensus of the need for a tailored approach to awareness raising and training on the transition to NAMs.^27^ More importantly, the process of convening groups to seek to understand their issues and concerns can assist in narrowing down the agenda of issues that need to be tackled.^28^ As discussed above, chemical safety assessment is risk-based and therefore value laden: such stakeholder discussions can help define the boundaries of a problem, to delimit science from the concerns of policy and law.

The roadmap also acknowledges the role of NGOs in the transition (having been instrumental in the ECI) and the importance, albeit the separate enterprise, of ‘engaging with the general public to bring about change.’^29^ Within the NGO community, however, there are diverging views that are very strongly held as to the readiness of NAMs to fully replace animal tests for human health endpoints.^30^ The reasoned engagement of NGOs will help the public understand the NAM capability for chemical risk assessment and the level of protection they offer. This is linked to the involvement of the public in the implementation phase and the roadmap notes that consumer choices may thus become better informed. This further extolls an expedited EU route to acceptance (discussed in the 'Validation and Standardisation' section, below), as a societal expectation to phase out animal testing has been demonstrated through, *inter alia*, pressure resulting in the cosmetics ban and the European Citizen’s Initiative that led to this roadmap. If awareness by consumers of the current extent of animal testing across the chemical industry can be heightened, then consumer demand for ethical products can become a driver for change. However, although the SWD does lay out forms of public engagement, such as citizen juries, by stating the reflections of the SAFE Consortium in an Annex III,^31^ it is not clear within the roadmap itself how the European Commission envisions these views inform implementation activity. This therefore leaves uncertainty as to how public engagement will unfold: the roadmap annex refers only to technical actions of the creation of an electronic hub and dashboard for both stakeholders and the general public. 32

This is also linked to a wider measure in the roadmap that may not only remedy the civil society division but also benefit the wider group of stakeholders. There is a growing need for more structured and targeted training, designed to cover the range of different audiences that might require NAM related education.^33^ There are different audiences with varied level of knowledge: industry practitioners, risk assessors, and managers who are already familiar with NAMs may benefit more from how NAMs should be used post-validation; whereas some other groups are less familiar with certain NAM technologies. One of the main caveats is funding and the roadmap does not say much about this in this context. Education policy is classified as a supporting competence, which limits the extent of EU action.^34^ This is further compounded by wider cost and funding issues, coupled with the broader questions as to how responsibility and delivery of training can be better structured and offered across the EU. No less important is the provision of training in different languages, as aligned with the EU multilingualism policy. Finally, the content of the educational provision will depend on the levels of training required (*i.e.*, formal education or staff development)^35^ as well as target audience.

### Regulatory culture

The roadmap provides a crucial signal to regulators from policymakers on the future direction of acceptable toxicology methods. Although REACH already requires that vertebrate animals are used as a last resort,^36^ regulatory culture has remained wedded to animal studies and some interpretations of REACH consider this is required.^37^ There is, therefore, an implementation problem. Beyond regulatory interpretation, however, some stakeholders, including regulators, lack familiarity with and therefore confidence in NAMs.^38^ Lack of confidence may stem from doubts about the utility of NAMs as an appropriate testing method, hampering acceptance of NAMs. The required level of confidence is not only dependent on the scientific maturity of NAMs but may also be a product of societal levels of risk appetite. It is by no means a homogenous landscape of chemical regulation: EFSA for example, are considered by some to be more open to NAM data.^39^ This does, however, mean there is an unevenness in regulatory progress on NAMs, which industry must contend with, and the implementation of the roadmap must also address. As mentioned earlier, providing training to regulators may be conceived as a technical endeavour, but without recognising that, at least in some quarters (such as under REACH), there is a perceived lack of confidence, efforts may underachieve if understanding as to where this lack of confidence stems is not tackled. While this may emanate from the extent to which the legal framework allows the exercise of regulatory discretion to accept NAM data, it is not the only answer, as evidenced by the slow progress made on adaptation to date, even when regulatory space is made for this.

To that end, the roadmap proposes ‘safe spaces’ and ‘regulatory exploration spaces’, which are both meaningful propositions to address shortcomings regarding expertise and confidence. The safe spaces provide a platform that could address any lack of trust that is present between industry and regulators, allowing both groups to understand the needs of users and their concerns about NAMs. Moreover, they provide an incentive to industry to share know-how and data. An important point to consider for safe space forums is regarding the level of information that would be shared within this space, as it may involve data for specific purposes, such as grouping or overall raw data from a variety of NAMs. That data must be fit for regulatory purposes and the ‘regulatory exploration spaces’ can test and accredit this. However, when setting up these spaces it is crucial to address industry concerns regarding data sharing and confidentiality and to consider legal frameworks to accommodate these. Industry will understandably seek some assurances that data from alternative approaches will not immediately prejudice a registration when protection levels, as discussed above, are yet to be clearly defined, and discussions should be receptive to expert, informed judgement.

Finally, as mentioned earlier, the roadmap identifies the need for ‘tailor-made’ training, which will not only benefit wider stakeholders but also EU and Member State agencies.^40^ Training is a huge endeavour for the implementation phase: achieving a skilled regulatory body that is adept at substance evaluation in the new NAM paradigm requires greater resource and energy than simply providing general awareness-raising activities.

### Validation and standardisation

Processes of validation and the development of OECD Test Guidelines (OECD TG) are often conflated.^41^ Although the roadmap does not directly tie these two, it demonstrates an acceptance landscape with many aspects: OECD TG, the EU Test Method Regulation, the International Organisation for Standardisation (ISO), and qualification. 42 Whereas validation is a process of ensuring the relevance and reliability of a method (typically at the EU level), an OECD TG is an international standardisation of a method that allows, in part, the ‘Mutual Acceptance of Data’ across OECD member states. A validated method need not become internationally standardised as an OECD TG before data can be submitted to regulators (see below regarding other routes to data submission under REACH, such as weight of evidence). Although updates to guidance, such as that published by ECHA, and amendment to the Test Method Regulation may denote regulatory acceptance of a method in principle,^43^ adherence to a validated method or an OECD TG does not guarantee that the data produced will be accepted by regulators; their substance evaluations will decide the regulatory relevance for the problem to which a method has been applied. As the EMA notes, regulatory acceptance ensures NAMs ‘are scientifically sound *and* suitable for use in regulatory decision making.’^44^

Although the roadmap refers to other means of assurance, such as ICH guidelines in pharmaceuticals and the standardisation of ISO and CEN, neither their individual values nor shortcomings are enlarged upon as alternative routes for chemicals. The SWD does, though, make clear that validation and OECD Test Guidelines are wrongly synonymised,^45^ and acknowledges that validation and regulatory acceptance currently take a long time.^46^ It is curious, however, that the main roadmap headlines are to make the validation process more efficient ‘by leading on the revision of OECD Guidance Document 34’^47^ and a milestone is that of reporting the number of test guidelines proposed at OECD,^48^ rather than considering an EU-level approach. Similarly, some activities in the SWD refer only to OECD TG (typically seen as a long, consensus process),^49^ rather than suggesting actions of EU-level validation.^50^ As noted above, although international harmonisation may be considered ideal, it is not necessarily an expedited path to replacement of animal studies within the EU.

EU legislative instruments such as REACH do allow for other routes towards regulatory acceptance, such as the Test Method Regulation (TMR),^51^ which gets only a passing reference in the roadmap.^52^ Although an international focus may be a sensible approach as other jurisdictions (such as the UK and USA) have also made various commitments to phase out animal testing, the expectation of formal validation raises the question of how future-proofed an international consensus approach is, especially considering the fast and dynamic pace of scientific innovation.

There is potential hope for a more adaptable approach in the roadmap by way of reference to the EU-NETVAL laboratory network and the PARERE network of Member States, on which a proposal is due for their optimisation by 2027. Working within existing legislative frameworks is likely to be more efficient than reform using the ordinary legislative procedure, and Directive 2010/63 on the Protection of Animals Used for Scientific Purposes already provides for the duties and tasks of the JRC (as the Union Reference Laboratory).^53^ These include: coordinating and promoting the development of alternatives; coordinating and participating in their validation; acting as a focal point for the exchange of information on NAMs; and promoting dialogue between stakeholder groups.^54^ With these networks, together with the TMR, and the role of the JRC, there is ample opportunity to take an EU-level view of validation and regulatory acceptance. This could be expedited using a point of broad consensus that we identified from our empirical research, whereby if a number of different approaches were to come to the same regulatory conclusion, then it would not be an unreasonable approach on which to base a regulatory decision.^55^ This EU-focused approach also does not preclude an international lens because, as the SWD notes, the international ICATM validation centre forum ‘discusses promising methods and evaluates their relevance’;^56^ the EU need therefore not progress in secret nor in a silo. Indeed, the use of non-standard research data (as may be compiled into a WoE submission) is mentioned in the SWD (the use of which was also a point of consensus in our stakeholder research) and the SWD even notes that there is OECD guidance on its use.^57^

Beyond representation at the OECD level, the primacy of the EU; its legislative instruments; and its leadership on the international stage should not be underestimated. Concurrent with the EU implementation phase, other jurisdictions are actively diverging by either concurring with the EU replacement policy (such as the UK and USA) or sticking with the status quo of animal studies. This means there may, for a time, be an uneven landscape of requirements, regardless of the route the EU takes. The importance of a NAM approach becoming ‘the standard’, and an international one at that, should not, however, be overlooked: beyond a process to have NAM data accepted within the EU, the use of the TMR approach could, in the initial implementation phase, be considered an interim route. The European Commission must ensure that any such alternative route not only demonstrates scientific credibility, but that the process has their formal backing as a recognised, transparent route with clear performance expectations and consistency that a method will provide. With authoritative support, the EU could grow recognition for the TMR and begin to shift it towards the recognition accorded to other standards. The development of a route to produce EU standards should not be confused with the role of OECD Mutual Acceptance of Data (MAD) across Member States, which can also be overstated, being as it is an acceptance of data rather than a decision and therefore is still not an assured route to market. But an opportunity that the optimisation proposal within the roadmap could expedite is the acceptance process within the EU, without affecting safety.

## The legal framework that underpins the roadmap

From the analysis above of the roadmap, it is clear that the phasing out of animal testing would benefit from a more extensive interpretation of legislation. However, the roadmap itself does also consider formal legal amendment. The format and current interpretation of legislation present a major barrier to the regulatory take-up of NAMs,^58^ and legislative change involves both time and political opportunity, which is needed also for guiding the interpretation of existing legislation to ensure the better use of NAMs. However, although the scope of the roadmap covers 15 ‘regulatory areas’ (Table 1), there is modest explicit reference to legal reform, either by amendment or more extensive interpretation. The roadmap acknowledges that ‘revisions to some legislation governing chemical safety assessment or guidance’ will be needed,^59^ but it remains largely silent as to what this will entail. The task of this roadmap is to lay out a policy rather than a legislative agenda, but the rigidity of existing the legislation is considered a major barrier to NAM acceptance. More might have been expected, therefore, on the process of reform to do more to indicate the direction of travel and to ensure buy-in for legal reform where this proves necessary to deliver regulatory change. This limitation may constitute a shortcoming both in terms of supporting the wide legislative remit of the roadmap and a bringing clarity as to how and when legislative change may be required. The understanding and interpretation of the law involves both hard and soft law instruments and both can assist in promoting NAMs, but the balance of these is a matter for careful calibration.

**Table 1:** Regulatory areas in scope of the EU roadmap.

1	REACH
2	Biocidal Products
3	Plant Protection Products (and maximum residue levels (MRL))
4	Food Improvement Agents (food additives, food enzymes and food flavourings)
5	Food Contact Materials
6	Feed Additives
7	Human Medicinal Products
8	Veterinary Medicinal Products (and MRLs for food-producing animals)
9	Medical Devices
10	Drinking Water
11	CAD and CMRD
12	Human Nutrition
13	Detergents
14	Classification, Labelling and Packaging
15	Water and Waste (identifying priority substances)

The roadmap refers to ‘regulatory areas’, of which 15 are identified. The roadmap also acknowledges the need for consistency with the ‘One Substance, One Assessment’ (OSOA) approach, towards which the Roadmap Steering Team will compile a regulatory needs report that will inform the European Commission OSOA expert group.^60^ While the OSOA Regulation (Regulation, 2025/2455 Establishing a Common Data Platform on Chemicals) lists 70 ‘Union legal acts,’^61^ this is perhaps not as large a discrepancy from the scope of the roadmap as first appears. Rather than listing legislation individually, the roadmap refers to ‘areas’ such as ‘Food improvement agents (food additives, food enzymes and food flavourings)’, which could encompass many other legislative instruments; whereas in OSOA, legislative instruments are listed separately and therefore more specifically, such as legislation on smoke flavourings and the addition of vitamins and minerals.^62^ Additionally, the roadmap is concerned with safety testing requirements of substances, which are typically at the point of risk assessment before chemical use and release, rather than for substance identification in surveillance and monitoring. The roadmap scope therefore also does not extend to legislative instruments addressed in the OSOA regulation such as those relating to, *inter alia*, urban wastewater treatment, agricultural nitrate release, the release of genetically modified organisms, polycyclic aromatic hydrocarbons in the air, and exposure to asbestos at work.^63^ Finally, there is a general acknowledgement of legislative instruments that are concerned with ‘Safe and Sustainable by Design’ in the roadmap, whereas eco-design measures are specified individually in OSOA. The risk assessment focus of the roadmap does not, however, consider this greater question of whether society needs to be using some of these substances at all.

The Annex of the roadmap document sets 2029 as the date by which the European Commission ‘will either adopt or make legislative proposals, as appropriate, to implement short term actions’ that will facilitate the 3R transition.^64^ The short-term actions that aim to replace animal testing are:

• Replacing *in vivo* with computational methods for acute oral toxicity and pharmacokinetic modelling;
• Replacing *in vivo* with *in vitro* for developmental neurotoxicity, malformations, embryofoetal lethality, pyrogenicity, and sensitisation.^65^

The short-term actions that aim to only reduce (rather than replace) animal testing are:^66^

• Waiving of *in vivo* based on historical experience regarding systemic toxicity in second species;
• Waiving studies based on target patient population on repeat dose toxicology for advanced cancers or severely debilitating or life-threatening diseases in human pharmaceutical development;
• Using *in vitro* or *in silico* models to predict drug-induced liver injury, pharmacokinetic parameters, cardiotoxicity, or immunotoxicity, and the use of virtual control groups in human pharmaceutical development;
• Study design optimisation, such as actions relating to dose groups; including additional endpoints in repeat dose toxicity studies; or using omics-enhanced studies rather than long-term *in vivo*.

One short-term action aims to refine (rather than replace or reduce) animal testing through using evident toxicity rather than lethality in acute toxicity studies.^67^

The short-term replacement actions appear relatively ambitious, with the roadmap addressing the replacement issue of the complex endpoint of developmental neurotoxicity, albeit by specifying *in vitro* rather than allowing for wider innovation. Otherwise, reductions (but not replacement) are primarily achieved via waiving, rather than the use of NAM data. However, although these are short term steps that the roadmap accepts may require legislative change, from closer scrutiny the actual task of the adaptation of legislation and guidance are only specified in four areas. These relate to three short-term actions and one mid-term:^68^

• Reduction or replacement for acute aquatic toxicity and for bioaccumulation;
• Reduction for chronic fish toxicity by clarifying fish testing requirements and waiving options;
• Reduction or replacement for endocrine disruption for environmental safety assessment to maximise information from *in vivo* testing.

Previous research on possible solutions to the better deployment of NAMs identified that directing NAMs to endpoints that are under-evaluated by animal studies, such as aquatic toxicity, may be a reasonable stepping stone to build confidence in NAMs, rather than directly replacing animals for human health effects.^69^ However, the roadmap is perhaps not as progressive in concrete steps as it first seems, with specific legislative actions mainly limited to environmental rather than human health endpoints, thereby arguably providing little direction regarding the replacement of animal studies for these health outcomes. Further, a risk of specifying these few areas is that a duplication of NAM development effort may ensue, creating bottlenecks of their own. In so doing, an unintended consequence might be that industry then falls back on animal testing in other endpoint areas.

There is, of course, a danger of promising too much and under-delivering, the risk of which the European Commission is likely to be aware. Additionally, the term of office of the European Commission only runs to 2029 and so there is a limited timeframe within which they can set out concrete measures to be undertaken. Even so, this should not be a seen as a limiting factor if the roadmap is to lead to its replacement destination. Just as a target focuses the mind, missing a date need not mean progress towards it was futile; a recycling target may be missed, but the efforts to recycle will still generate saved resources. Similarly, without a date the formation of milestones and indicators becomes problematic, if it is not clearly discernible what the end point is to be: it is difficult to plan a route without a location. Ultimately, however, the roadmap aspires to transition because it provides a direction of travel and the ultimate aim of replacement, and perhaps this will be sufficient to encourage scientists and innovators to flexibly fill in the gaps. Fixing timelines for scientific advance is not easy, but accountability for timely progress remains crucial.

Nevertheless, legislative change is constrained by focusing on limited short-term actions, and the ability of the roadmap to build in flexibility for future innovation is also limited. While the mid-to-long term actions prescribed by the roadmap do not preclude science from developing and attempting to be used, without policy and legislative planning in the roadmap itself there may be a continuing reluctance to submit data from NAMs for other endpoints. Additionally, the focus of the roadmap on validation before regulatory acceptance is likely to string timelines out even further, particularly without any specified phase out or milestone dates. Therefore, it is likely that the transition will continue to slip, without concrete indications against which progress can be measured.

In support of innovation and for registrants who would prefer to utilise NAMs, the roadmap acknowledges the use of weight-of-evidence (WoE) as a mid-term action as a framework to address genotoxicity, carcinogenicity, reproductive toxicity, endocrine disruption, and nanomaterial assessment.^70^ Although this is a general statement, without mention of concrete measures to take forward, most of the 15 legislative instruments within the scope of the roadmap do have the capacity to accept NAM data, especially through WoE.^71^ The Plant Protection Products regulation extensively encourages animal studies (which will require legislative change), yet, better use of soft law instruments such as guidance would be very beneficial for registrants to utilise the provision for NAMs elsewhere, especially by reliance on WoE. More detailed soft law guidance may allow greater flexibility for innovation that is currently developing or is yet to develop, rather than changing legislation through the ordinary legislative procedure. However, there may be little appetite for law reform and concerns regarding the perception of competence overreach if ECHA, for example, were to provide more specific guidance around NAMs. Having said that, this policy roadmap could be considered as laying the groundwork for precisely such a revision of guidance around those mid-term action endpoints, based on WoE, to address human health outcomes as the areas of greatest animal use.

Related to discussions on the EU legal framework that underpins the roadmap is the analysis of cases of the ECHA Board of Appeals and the case law of EU courts (including Court of Justice of the European Union (CJEU) and General Court), which is often overlooked as a part of the wider discussion on the gradual phasing out of animal testing. Although EU case law is not formally recognised as a source of law, it is of great significance in enforcement and interpretation of EU chemicals law, in particular the REACH commitment to use animals as a last resort. The roadmap only briefly mentions the ‘last resort’ principle with regards to the list of measures under each pillar of the roadmap. Under Pillar I, the creation of regulatory exploration spaces and safe spaces should strengthen use of this principle.^72^ To that end, the European Commission will work with ECHA and the Member State Committee (MSC) to ‘strengthen their efforts to accept animal testing proposed by registrants only as the last resort, in line with the objective of this roadmap.’^73^ However, this does not address the more inherent problem relating to how EU courts are interpreting the ‘last resort’ principle. Courts are by nature very cautious in assessing scientific evidence and its reliability, which leads to deference to scientific decision-making by regulatory bodies. This is coupled with the reliance on the precautionary principle, which unfortunately encourages and sometimes requires the use of animal testing where the evidence from non-animal testing is seen as less certain.^74^ These approaches erode the application of the last resort imperative as stipulated by REACH, which, if the roadmap is to succeed, must make animal testing just that: the last resort. Any appeals' process has to recognise this and adjudicate accordingly, and thus the court and appeals systems should also be engaged.

Analysis of EU legislation and case law governing chemicals and their capacity to accommodate NAMs allows us to observe more clearly those actions that are not within the spirit of the legislation (*i.e.*, vertebrate animal studies). The presence of adaptations such as WoE, in particular, provides a future-proofed approach that enables science and technology to develop (supported by soft law guidance), without requiring onerous legislative updates to accommodate specific methods. This framing more strongly acknowledges the place of NAMs instrumentally in legislation. Finally, the modest reference to the ‘last resort’ principle and lack of reference to courts in the roadmap does, however, question the inclusion of all stakeholders that are key in implementing this principle. For example, in the US an ‘Genomics for Judges’ educational course prepares judges for dealing with cases involving sequencing data and technologies, aiding them to understand such data and evidence.^75^ The experiences of registrants in the courts contributes to the perceptions within the community (and the Board of Appeal Digest may also be considered a source of soft law guidance), and thus ensuring the courts might feel confident reviewing both contested NAM data (rather than resorting to animal studies out of familiarity) and deciding cases defended by regulators regarding the potential over- (or even under-) regulation of substances, is important for rounding the circle of NAM acceptance. The consideration within the roadmap of awareness raising in the court system would have ensured a more comprehensive strategy for replacing animal studies.

## Transition trajectory

A final point of note in this analysis of the roadmap relates to that ultimate destination of the replacement of animals in testing. A footnote of the roadmap states that ‘the roadmap therefore considers replacing the use of non-protected life stages and invertebrates as a longer-term goal.’^76^ This has implications for many NAM developers who are currently providing methods and data that are based on those life stages. Directive 2010/63, after all, protects life stages that are:^77^

• Live non-human vertebrate animals, including
  ○ Independently feeding larval forms; and
  ○ Foetal forms of mammals as from the last third of their normal development;
• Live cephalopods.

The 2010/63 Directive recognises ‘the capacity of animals to sense and express pain, suffering, distress, and lasting harm,’^78^ and therefore establishes measures relating to, *inter alia,* replacing and reducing these animals in procedures, and for their breeding and care, to protect them.^79^ To that end, some NAM developers have developed approaches that utilise those animals and life stages for which there is not the scientific evidence to demonstrate that they feel pain. A benefit of this is that the responses of these live organisms can still be measured, rather than complete reliance on computer modelling or interpolation regarding chemical structure.

The phasing out of non-protected life stages under the roadmap, as well as vertebrate animals, demonstrates a divergence in policy - invertebrates may be used in science, but not in the longer term for regulatory decision-making within the scope of the roadmap. These organisms could theoretically continue to be used in-house, as some currently are, but any outputs should not later be used to support regulatory decisions.

This move may be a welcome reflection of international and public perception reality. Such distinctions of protected animals are not made in the USA, and the internationally recognised ‘leaping bunny’ logo used in cosmetic and household products relates to ‘non-human animals in the kingdom “Animalia.”’^80^ However, while a noble pursuit, this policy has implications for science innovation, which raises the question as to whether this aim should be more obviously stated, so as to better inform NAM investment decisions, and indeed for EU funding to be similarly redirected. The European Commission may consider this ‘longer-term goal’ as being so far in the future that the current use of invertebrates will be a true stepping stone to full replacement. However, there is a risk that this policy announcement leads to this section of NAM science (those utilising life stages not protected by Directive 2010/63) still not being laid as a stepping stone at all, due to fears (genuine, or otherwise) that such investment and acceptance will be a distraction along the route to true replacement. The course to be taken then suffers from the same wranglings as can be observed in climate change mitigation and adaptation debates and the energy transition: does investment in one option detract from another, especially if transition speed is an imperative?

## Conclusion

The roadmap was a long-awaited strategy document that represents an important policy step in acknowledging the value of NAMs in transiting to non-animal testing. It recognises major barriers in this journey and the need for more robust implementation measures. However, the roadmap and the more extensive SWD are still predominantly focused on the technical aspects of the transition, leaving the social nature of barriers to acceptance (such as consideration as to how engagement might be encouraged) still to be addressed in detail during the implementation phase. Although these documents acknowledge, for example, the importance of engaging with the public for democratic resilience and to build trust in science, it provides little in the way of steps to achieve this.

This paper has set out how the successful implementation of the roadmap requires the agreed definition of acceptable levels of protection and risk within the new paradigm to which NAMs are to be applied. The subsequent clarity will benefit NAM data users and NAM developers. This consensus journey may be complex but ensuring a comprehensive range of stakeholders are involved will provide depth and aid legitimacy. The ‘Catalogue of Transitional Initiatives’ will then be a valuable resource for raising awareness of current project developments on NAMs and of use in identifying knowledge gaps. This also requires careful consideration, however, of how the Catalogue can be developed in a comprehensive form and also be kept up to date.

Awareness raising and targeted, multilingual training on NAMs, tailored to needs, is vital to raise confidence and competence but is known to be hampered by costs, which is not addressed in the roadmap, nor the wider social facets required to build confidence. While the safe spaces introduced by the roadmap may assist, such spaces are fraught with their own concerns, such as on confidentiality and data sharing, which must be addressed in detail. Additionally, although alternative routes to assurance are available and acknowledged, the roadmap focuses on OECD Test Guidelines. We can hope that the optimisation proposal for EU-NETVAL and PARERE favourably considers the greater utility of existing structures, such as the JRC and the TMR, as a route to the expedited regulatory acceptance of NAMs and, in particular, the recognition by the European Commission of the TMR as a home for EU standards.

While the roadmap does identify a large number of stakeholders that would be part of the implementation phase, it does not make reference to the ECHA appeals and EU judicial system, which plays an important role in providing interpretation to EU chemical law and its enforcement, in particular the well-known obligation under Article 25 of REACH to use animal testing as a last resort. Legislative instruments do largely have the capacity to accept human and environmentally relevant NAM data with some exceptions, such as PPP. Although the roadmap identifies the need for legal reform, the nature of that reform is not explicitly elaborated. The development of soft law guidance to aid the use of this adaptation by registrants would encourage the application of innovation, rather than relying on slower legislative routes. Without this, NAM innovators may regrettably focus on the narrow endpoints selected in the roadmap, leaving other endpoints under-developed and therefore remaining subject to animal studies.

The roadmap identifies three guiding pillars and a list of short-, mid-, and long-term actions and opportunities. As this is a strategic document, the plans on legal reform may come later in a forthcoming European Commission annual work programme, which might set out new policy and legal initiatives to be brought forward. However, in failing to stipulate hard phase out dates and no real milestones, this strategic approach means it will be difficult to measure or indicate progress. The emphasis of the roadmap on, for example, validation as an expectation prior to regulatory uptake, risks becoming a pre-occupation that may stretch the already hazy timeframe. Even the trajectory of the phasing out transition may become problematic for some NAM innovators in light of the longer-term goal of the roadmap to replace the use of the currently non-protected life stages and invertebrates in testing. This, coupled with the limited remaining term of this Commission restricting its ability to set firm dates in the future, means that achieving full animal replacement may be delayed longer than some stakeholders had hoped.

That being said, the publication of the roadmap is in itself a great achievement for the European Commission, through what would have been a hugely challenging task. The involvement of a wide array of stakeholders in workshops and discussions that informed and shaped it lends legitimacy to this policy, and, regardless of the path chosen, the response from the European Commission to concerted societal pressure has finally resulted in a clear intention that, across multiple industry sectors, animal testing will ultimately be phased out. We welcome the implementation phase and hope that the social elements of how the roadmap activities will be carried out is considered thoughtfully, to fully realise the potential of this strategy, and reflecting the effort that has gone into its development.

## CRediT authorship contribution statement

**Aleksandra Čavoški:** Writing – review & editing, Writing – original draft, Investigation, Conceptualization. **Laura Holden:** Writing – review & editing, Writing – original draft, Investigation, Conceptualization. **Robert G. Lee:** Writing – review & editing, Writing – original draft, Investigation, Conceptualization.

## Declaration of competing interest

The authors declare that they have no known competing financial interests or personal relationships that could have appeared to influence the work reported in this paper.

## Data Availability

Data will be made available on request.

## Footnotes

65 European Commission at n2, p22.

## Footnotes

1 See Article 25 of the Regulation (EC) No 1907/2006, 2006 and Article 4 of the Council Directive 2010/63/EU, 2010 (‘Directive 2010/63’).

2 European Commission (2026a), p. 1.

3 See also Article 24 of the Treaty on the Functioning of the European Union (TFEU) (2016), (‘TFEU’); and Regulation (EU), 2019.

4 European Commission at n2, p. 3.

5 Čavoški et al. (2024a); Čavoški et al. (2025a); Bearth et al. (2025).

6 Čavoški et al. (2025a), at n5.

7 European Commission (2026b).

8 European Commission at n2, p. 5.

9 Čavoški et al. (2025c), pp. 22, 41.

10 European Commission at n2, p. 5.

11 European Commission at n7, p. 31.

12 Ibid.

13 An ECHA presentation on 11 September 2025 did touch on this: ECHA, 2025.

14 European Commission at n7, p. 32.

15 Čavoški (2024) at n5, p. 36.

16 REACH at n1.

17 Such as the International Standards for Phytosanitary Measures (ISPM) (2008).

18 European Commission at n7, p.34 and p. 35 at fn 44.

19 European Commission at n7, p. 36.

20 European Commission at n2, p. 6.

21 Čavoški et al. (2025d).

22 European Commission at n7, p. 197.

23 European Commission at n2, p. 6, s3.1.4.

24 European Commission at n2, p. 5.

25 European Commission at n7, p. 36.

26 European Commission at n2. P. 6.

27 Čavoški et al. (2024b).

28 Ibid.

29 European Commission at n2, p. 14.

30 Čavoški et al. (2025b).

31 European Commission at n7, p. 245.

32 European Commission at n2, p. 20.

33 Čavoški et al. (2024b) at n27; and European Commission at n2, p. 14.

34 TFEU at n3, Article 6.

35 Čavoški et al. (2024b) at n27.

36 REACH at n1, Article 25.

37 Čavoški et al. (2024b), at n5.

38 Čavoški et al. (2024b), at n5.

39 Čavoški et al. (2024b), at n5, p. 18.

40 European Commission at n2, p. 14.

41 Čavoški et al. (2024b), at n5.

42 European Commission at n2, pp. 6-7, s3.1.5.

43 Regulation (EC) No 440/2008, 2008 (‘TMR’).

44 European Medicines Agency, 2026, emphasis added.

45 European Commission at n7, p. 201.

46 European Commission at n7, p. 203.

47 European Commission at n2, p. 9.

48 European Commission at n2, p. 19, action 8.

49 European Commission at n7, p. 202; Čavoški et al. (2024b), at n5.

50 European Commission at n7, p. 56: regarding TK and ADME models, ‘In the short term: development of OECD Test Guidelines for the generation of metabolic clearance data’.

51 REACH at n1, Article 13; ‘TMR’ at n43.

52 European Commission at n2, p. 7.

53 Directive 2010/63 at n1, Article 48.

54 Directive 2010/63 at n1, Annex VII.

55 Čavoški et al. (2024b) at n27.

56 European Commission at n7, p. 202. The UK is also looking to set up its own validation centre, UKCVAM.

57 European Commission at n7, p. 205; and Čavoški et al. (2024b) at n27.

58 Čavoški et al. (2025d) at n5; Čavoški et al. (2024b) at n5; Schiffelers et al. (2012); Fentem et al. (2021).

59 European Commission at n2, p. 5.

60 European Commission at n2, pp. 5, 7.

61 Regulation (EU) 2025/2455, 2025, Annex I, Part I.

62 Ibid, No.15 and No. 26.

63 See Regulation, 2025/2455 at n52.

64 European Commission at n2, p. 17.

66 Ibid.

67 European Commission at n2, p. 23.

68 European Commission at n2, pp. 24-25. See the ‘Overview’ provided in Table 1 of the roadmap Annex and the detail provided in the ‘Means to achieve it’ column of Table 3 of the Annex.

69 Čavoški et al. (2024b) at n27.

70 European Commission at n2, p. 23.

71 Čavoški et al. (2025d) at n9.

72 European Commission at n2, p. 9

73 European Commission at n2, p. 19

74 Čavoški et al. (2026).

75 University of Illinois Urbana-Champaign, 2026.

76 European Commission at n2, p. 3, fn 18.

77 Directive 2010/63 at n1, Article 1(3).

78 Directive 2010/63 at n1, recitals 6, see also recital 8 regarding cephalopods.

79 Directive 2010/63 at n1, Article 1(1).

80 Čavoški et al., at n65.
